# Disseminated Histoplasmosis Mimicking Macrophage Activation Syndrome in a Patient With Rheumatoid Arthritis

**DOI:** 10.7759/cureus.53723

**Published:** 2024-02-06

**Authors:** Ashwin Jagadish, Shahnawaz N Notta, Nasir Notta, Muhammad Abdur Raafey, Gerald Falasca

**Affiliations:** 1 Internal Medicine, East Tennessee State University James H. Quillen College of Medicine, Johnson City, USA; 2 Pathology and Laboratory Medicine, East Tennessee State University, Johnson City, USA; 3 Rheumatology, Medical Specialists of Johnson City, Johnson City, USA

**Keywords:** immunosuppression, hemophagocytic lymphohistiocytosis (hlh), macrophage activation syndrome, disseminated histoplasmosis, rheumatoid arthritis

## Abstract

Infections caused by *Histoplasma capsulatum* typically do not produce symptoms. However, in individuals who are immunocompromised, progressive disseminated histoplasmosis may occur. A 67-year-old female, with lengthy history of immunosuppression due to management of rheumatoid arthritis, reported a two-month history of fatigue, headaches, and intermittent fevers following hip surgery. Due to the concern for macrophage activation syndrome and hemophagocytic lymphohistiocytosis, a bone marrow biopsy was performed. However, the results indicated the presence of *Histoplasma*, which was supported by the presence of *Histoplasma* antigens in the serum and urine, *Histoplasma* antibodies in the serum, positive (1,3)-beta-D-glucan results, and fungal blood cultures. The patient initially received amphotericin B, but it was switched to itraconazole due to adverse effects. The patient remains on itraconazole therapy and follows as an outpatient with an infectious diseases specialist.

## Introduction

Rheumatoid arthritis (RA) is an autoimmune disorder that can lead to chronic joint inflammation, damage of the joints, cartilage loss, and bone damage [1]. RA is influenced by both genetic and environmental factors [1]. Treatment can include disease-modifying antirheumatic drugs, non-steroidal anti-inflammatory drugs, corticosteroids, nonbiologic disease-modifying antirheumatic drugs, tumor necrosis factor alpha inhibitors, rituximab, abatacept, interleukin 6 inhibitors, or Janus kinase inhibitors [1]. Many of these medications are associated with an increased risk of infections [1].

*Histoplasma capsulatum* (*H. capsulatum*) is prevalent across the globe, with more than half the population possibly living in a region where it is endemic [2]; in the United States, the most common locations are the Ohio and Mississippi river valleys [3]. *Histoplasma capsulatum* has the ability to remain dormant for a period of time and reactivate if cell-mediated immune function declines, such as through medications that suppress the immune system [4]. Presentations of histoplasmosis include acute pulmonary histoplasmosis, chronic pulmonary histoplasmosis, and progressive disseminated histoplasmosis [2]. *Histoplasma* infections often do not result in symptoms; however, immunocompromised individuals are at a higher risk of disseminated presentation [2]. Hepatosplenomegaly can be seen in approximately 50% of patients with disseminated histoplasmosis, although the condition can impact any organ [4]. Many times, an identifiable source is not found in individuals with disseminated histoplasmosis [4]. Without treatment, progressive disseminated histoplasmosis can be fatal [2].

## Case presentation

A 67-year-old female with longstanding RA presented to an emergency department in Tennessee due to weakness accompanied by a ground-level fall. In addition, she reported a two-month history of fatigue, headaches, intermittent fevers, and night sweats that initially began after a right total hip arthroplasty. Her medications included tocilizumab, most recently administered one week prior, and prednisone, administered daily. Adalimumab was discontinued four months prior due to inefficacy, while methotrexate was discontinued prior to surgery. In the past, she received etanercept for several years.

**Figure 1:**
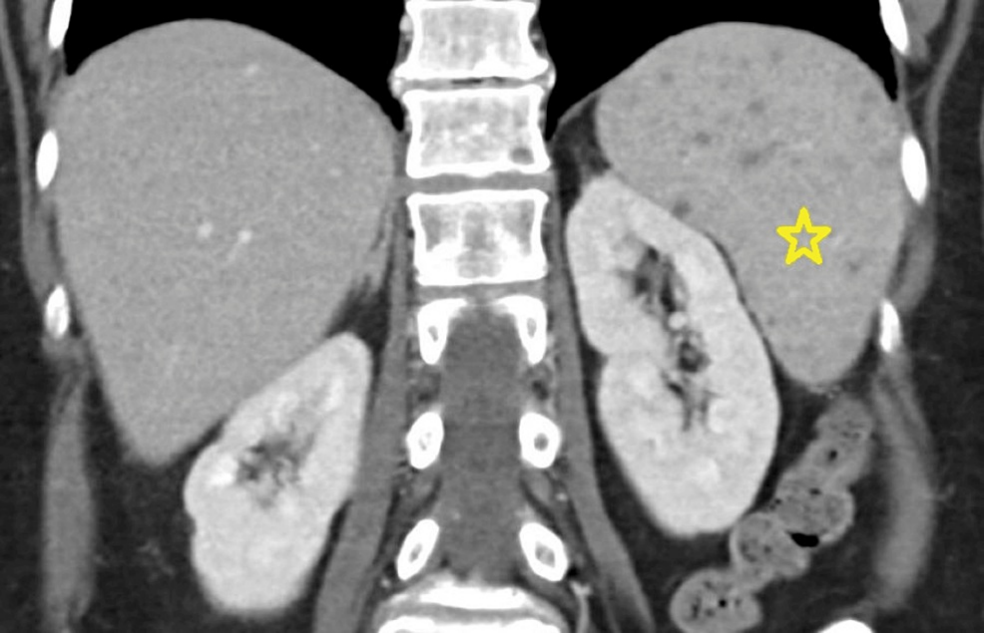
Computed tomography of the spleen The star indicates an enlarged spleen containing several hypodense probable complex cysts or hemangiomas.

In the emergency department, her temperature was 97.2°F, heart rate was 85 beats per minute, blood pressure was 102/54 mmHg, oxygen saturation was 98%, and respiratory rate was 18 breaths per minute. Her laboratory evaluation was notable for hemoglobin of 11.1 g/dL (normal range: 12.4-15.2 g/dL), ferritin level of 3,120 ng/mL (normal range: 10-291 g/dL), platelet count of 134,000 cells/µL (normal range: 150,000-450,000 cells/µL), and sodium of 124 mmol/L (normal range: 137-145 mmol/L). The most recent outpatient ferritin level obtained in the month prior to hospitalization was 5,085 ng/mL. She was admitted for symptomatic hyponatremia. Computed tomography (CT) of the abdomen and pelvis demonstrated a prominent liver, along with an enlarged spleen containing several hypodense probable complex cysts or hemangiomas (Figure 1). CT imaging of the head did not reveal abnormalities. Testing for immunoglobulin M antibodies against Epstein-Barr virus and cytomegalovirus revealed no abnormalities. The most recent QuantiFERON TB Gold, completed within the past year, was negative. Evaluation of immunoglobulins A, G, and M revealed normal values.

**Figure 2:**
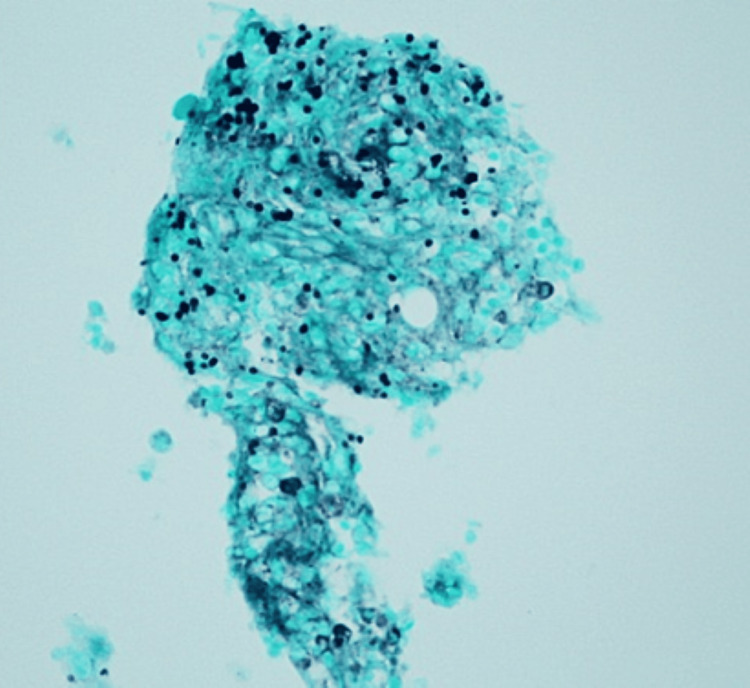
Grocott-Gomori methenamine silver stain

**Figure 3:**
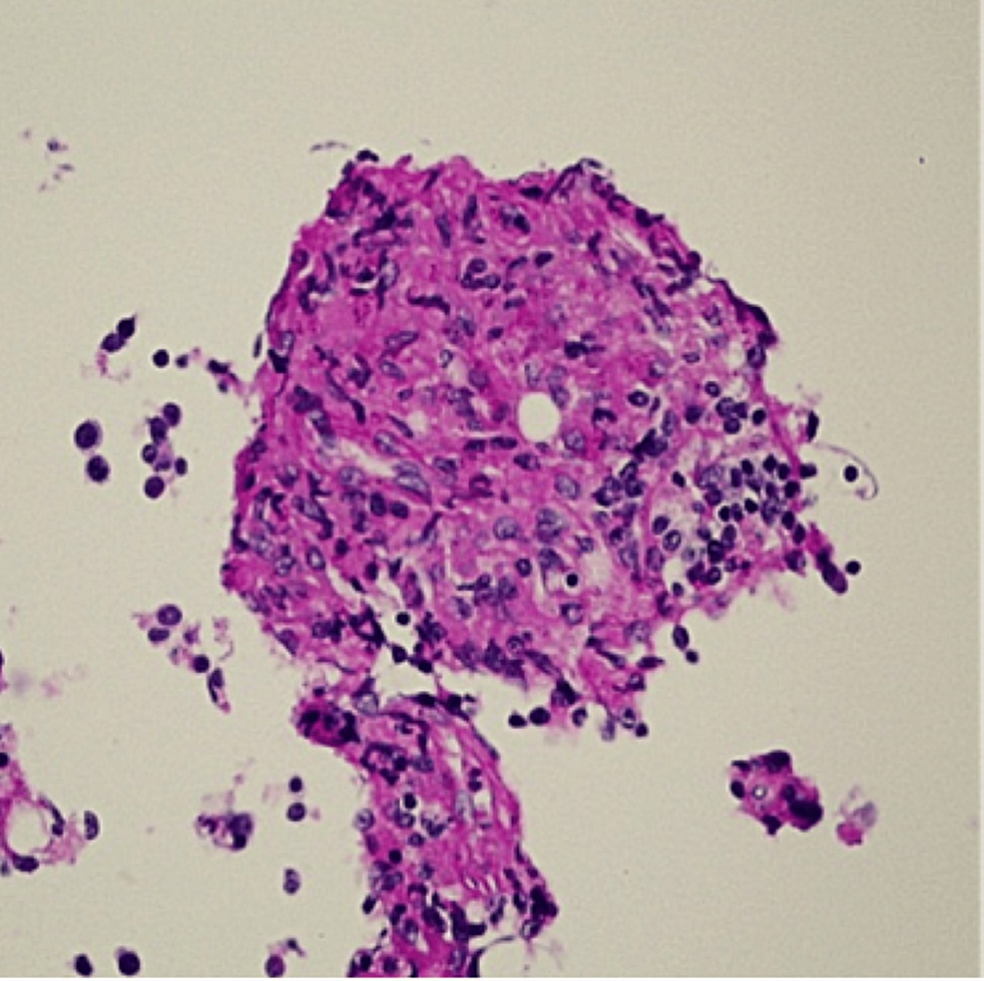
Periodic acid-Schiff stain

Her temperature fluctuated over the next few days, reaching 102.4°F on the fifth hospital day. Blood cultures obtained at admission revealed no organismal growth, while chest radiograph and urinalysis showed no evidence of infection. The only antibiotic provided was 1 g of intravenous ceftriaxone on the day of presentation to the hospital. Given her findings and rheumatologic history, there was concern for hemophagocytic lymphohistiocytosis (HLH) and macrophage activation syndrome (MAS). Her platelet count was 113,000 cells/µL, fibrinogen level was 220 mg/dL (normal range: 172-510 mg/dL), and triglycerides were 234 mg/dL (normal range: 0-149 mg/dL). Echocardiogram did not reveal any valvular vegetations. On the sixth hospital day, a right iliac crest bone marrow biopsy was performed to evaluate for potential HLH and MAS. The following day, CT of the chest revealed a nonspecific nodular pleural-based opacity in the left lower lobe. By then, the biopsy results revealed non-necrotizing granulomas with yeast forms consistent with histoplasmosis; these were identified on Grocott-Gomori methenamine silver (Figure 2), periodic acid-Schiff (Figure 3), Prussian blue, and routine stains. There was no bone marrow evidence supporting MAS or HLH.

The patient was subsequently started on intravenous amphotericin B at a rate of 3 mg/kg every 24 hours. Lumbar puncture and magnetic resonance imaging of the brain did not suggest histoplasmosis of the central nervous system. A meningitis/encephalitis panel by polymerase chain reaction, evaluating the cerebrospinal fluid for *Escherichia coli* K1, *Haemophilus influenza*, *Listeria monocytogenes*, *Neisseria meningitides*, *Streptococcus agalactiae*, *Streptococcus pneumoniae*, cytomegalovirus, herpes simplex virus (HSV) 1, HSV 2, human parechovirus, varicella zoster virus, and *Cryptococcus gatti/neoformans*, was negative. Testing for human immunodeficiency virus was negative as well. However, *Histoplasma* antigens were detected in the serum and urine; in addition, *Histoplasma* antibodies were identified in the serum. Also, (1,3)-beta-D-glucan (Fungitell©, Associates of Cape Cod, Inc., East Falmouth, MA, USA) was >500 pg/mL (positive value: ≥80 pg/mL). These results supported a diagnosis of disseminated histoplasmosis. After seven days of amphotericin B, the therapy was discontinued due to an elevated aspartate aminotransferase (AST) level of 147 U/L (normal range: 14-36 U/L) and alanine aminotransferase (ALT) level of 248 U/L (normal range: 0-34 U/L). The patient was subsequently started on oral itraconazole therapy with a dose of 200 mg thrice daily for 3 days, followed by 200 mg twice daily for one year. After that, it was recommended that she take itraconazole 200 mg once daily as prophylaxis for the remainder of her lifetime due to the potential need for immunosuppressive therapy for her RA. On the same day as the change in medications, she was discharged with instructions to follow up as an outpatient.

In the following month, a fungal blood culture obtained during the hospitalization grew presumptive *Histoplasma* species; a formal diagnosis of *H. capsulatum* was confirmed the subsequent month. Five months later, a CT of the chest, abdomen, and pelvis demonstrated no acute findings other than a stable pleural density. The spleen did not demonstrate any significant findings. Two months later, the patient was evaluated in the rheumatology clinic and appeared stable. Her only current rheumatologic medication is hydroxychloroquine. She reported continuing itraconazole therapy and following up with an infectious diseases specialist, as directed.

## Discussion

Our patient had an autoimmune condition, RA, which was accompanied by hepatosplenomegaly, hyperferritinemia, reduced platelet levels, persistent fevers, and hypertriglyceridemia. As a result, we were considering MAS and HLH as our leading differential diagnoses.

MAS is a serious complication of rheumatologic disorders that can be fatal [5]. Some signs of MAS include enlargement of the liver and spleen, chronic elevated fevers, and lymph node involvement [5]. Laboratory testing may demonstrate reductions in leukocytes, platelets, fibrinogen, and albumin; there may also be increases in AST, ferritin, and lactate dehydrogenase [5].

HLH has primary and secondary types, of which the secondary type is more common in adults [6]. Autoimmune conditions are known to be associated with secondary HLH [6]. According to the 2004 criteria, a diagnosis of HLH can be made if five of the following criteria are met: at least two cytopenias, enlarged spleen, elevated triglycerides with or without decreased fibrinogen, fever, hemophagocytosis demonstrated through biopsy, ferritin level in excess of 500 ng/mL, natural killer cell function, which is reduced or not present, and serum interleukin-2 receptor alpha chain ≥ 2,400 U/mL [6].

Diagnosis of *Histoplasma* infection may involve antibody testing, latex agglutination, Western blot, enzyme immune assays, and antigen testing in the serum or urine [2]. Testing for antigens may be more effective than other methods in individuals with decreased immune function [2]. Identifying *Histoplasma* in the bone marrow has been seen in fewer than 4% of cases [7]. A bone marrow biopsy would not have been performed in our patient’s case if the differential diagnosis of HLH was not being considered. The treatment of choice for progressive disseminated histoplasmosis is two to four weeks of amphotericin B followed by one year of itraconazole [2].

*Histoplasma* has been noted in individuals receiving immunosuppressive therapies such as methotrexate, prednisone, leflunomide, adalimumab, infliximab, and etanercept [8]. In addition, histoplasmosis has been seen in a patient receiving tocilizumab [9]. Over the years, our patient received immunosuppressive agents, which likely increased her susceptibility to this condition.

## Conclusions

*Histoplasma* is present across the globe, but infections may be asymptomatic. Patients with autoimmune conditions receiving immunosuppressive therapies are at an increased risk of developing disseminated histoplasmosis, which can be fatal if untreated. Features sometimes overlap with those of MAS or HLH. It is important for the clinician to consider histoplasmosis among the differential diagnoses in patients with fevers of unknown origin so that the patient can be treated.
